# The combined application of Mini-CEX and Check-list Scales in enhancing clinical competence among emergency and critical care residents: a comparative study

**DOI:** 10.3389/fmed.2025.1492206

**Published:** 2025-07-11

**Authors:** Xiao-guang Cao, Jia-xin Hu, Huang-chong Jian, Xiong-feng Zhu, Huadong Meng, Min Shao

**Affiliations:** ^1^Department of Critical Care Medicine, The First Affiliated Hospital of Anhui Medical University, Hefei, Anhui, China; ^2^Department of Emergency Medical Center, The First Affiliated Hospital of University of Science and Technology of China (Anhui Provincial Hospital), Hefei, Anhui, China; ^3^Huashan Hospital, Fudan University, Shanghai, China; ^4^Suzhou Hospital of Anhui Medical University (Suzhou Municipal Hospital of Anhui Province), Suhou, Anhui, China; ^5^The Third people’s Hospital of Hefei, Hefei, Anhui, China; ^6^Department of Emergency Intensive Care Unit (EICU), The Third Affiliated Hospital of Anhui Medical University (The first people’s Hospital of Hefei), Hefei, Anhui, China

**Keywords:** Mini-CEX, Check-list, emergency and critical care, medical education, medical training

## Abstract

**Objectives:**

This study aims to explore the value of integrating the Mini-Clinical Evaluation Exercise (Mini-CEX) and Check-list Scales in the training of emergency and critical care residents. The study evaluates the effectiveness of these tools in enhancing clinical diagnostic skills, improving teaching outcomes, and optimizing clinical processes.

**Methods:**

This study included 199 emergency and critical care residents who completed their training between January 2018 and April 2024. A paired study design was employed to evaluate the effectiveness of the combined use of the Mini-CEX and Check-list Scales. Initially, all participants used the Mini-CEX to assess their performance during clinical diagnosis and treatment (control group). After training with the Check-list Scales, the same participants underwent a second assessment using the Mini-CEX scale (experimental group). Data were analyzed using various statistical methods, including chi-square tests for categorical data, *t*-tests for normally distributed data, rank-sum tests for non-normally distributed data, and receiver operating characteristic (ROC) curve analysis to evaluate diagnostic performance.

**Results:**

The combination of Mini-CEX and Check-list significantly improved clinical competencies across several domains. In the control group, the overall failure rate was 2.513%, the pass rate was 70.352%, and the excellence rate was 27.136%. In contrast, the experimental group showed a reduction in the failure rate to 0%, with a pass rate of 19.598% and an excellence rate of 80.402%. The Mini-CEX scores in the experimental group were significantly higher than those in the control group (*p* < 0.001), with marked improvements in individual competencies, especially in clinical judgment and overall clinical competence. However, the experimental group had a longer diagnosis and treatment time compared to the control group (66.985 ± 9.126 min vs. 52.387 ± 7.635 min, *p* < 0.05). Correlation analysis revealed significant associations between various parameters before and after using Check-list tools, indicating improved diagnostic efficiency and clinical skills. The correlation between total score and components such as medical interviewing skills, physical examination skills, and overall clinical competence was notably stronger after the use of the Check-list (*p* < 0.05). ROC curve analysis demonstrated that all factors had good diagnostic performance, with the lowest being consultation/advice and communication skills [area under curve (AUC) 0.716, 95% CI: 0.680–0.752) and humanistic qualities/professionalism (AUC 0.733, 95% CI: 0.696–0.770), and the highest being clinical judgment (AUC 0.844, 95% CI: 0.813–0.875) and organizational skills/efficiency (AUC 0.815, 95% CI: 0.782–0.848).

**Conclusion:**

The integration of the Mini-CEX and Check-list significantly enhances the diagnostic and clinical skills of emergency medicine residents. This combined approach addresses the limitations of traditional training methods and provides an effective model for improving medical education and the quality of care for critically ill patients.

## 1 Background

As society advances and the economy grows, the global burden of disease continues to escalate, imposing greater demands on the diagnostic and therapeutic capabilities of healthcare professionals, particularly in China (1–3). In response, the cultivation of clinical skills has become a central focus in global medical education (4). However, traditional teaching methods are increasingly inadequate for the evolving needs of modern healthcare systems (5, 6), making the effective delivery of clinical education a critical challenge. This issue is especially acute in China, where the working environment in emergency and critical care units is notably more complex than in many developed countries (7, 8). Due to strained doctor–patient relationships and difficult clinical settings, highly skilled or experienced healthcare professionals are often reluctant to work in such departments (9, 10). As a result, junior residents—despite their limited clinical experience and decision-making capacity—are frequently placed in these high-stakes environments (11, 12).

To address the gap between training needs and clinical demands, competency-based evaluation tools such as the Mini-CEX and the Check-list have been widely introduced. Mini-CEX, recommended by the American Board of Internal Medicine, is commonly used to assess residents’ core clinical competencies and is integrated into routine educational practice (13). It provides a comprehensive assessment of communication skills, professional competence, and clinical reasoning. Its flexibility across diverse clinical contexts and capacity to deliver formative feedback make it well-suited for longitudinal competency tracking, although its effectiveness may vary depending on evaluator consistency and clinical setting (14–16). In contrast, Check-lists are structured evaluation tools developed from clinical guidelines and expert consensus. They promote diagnostic standardization and procedural adherence, thereby enhancing safety and clinical efficiency (17–19). Nevertheless, Check-lists often underemphasize non-technical competencies such as humanistic care, communication (20), and critical thinking, and they may lack formative feedback mechanisms (21). Furthermore, widespread medical disputes (22), cost-control reforms (23), and workforce burnout (24) have compounded the challenges of cultivating clinical and humanistic competencies in China’s emergency settings. These institutional constraints limit not only the effectiveness of clinical teaching but also residents’ capacity for communication, empathy, and timely decision-making.

Although both the Mini-CEX and Check-lists have demonstrated considerable educational value when applied independently (25, 26), their respective roles in this study are clearly defined: the Check-list functions as the structured instructional intervention, while the Mini-CEX serves as the evaluation tool to measure its impact. While each tool has been widely used in isolation, few studies have systematically compared the effectiveness of Mini-CEX when integrated with Check-list-based training versus when paired with conventional clinical instruction, particularly in emergency and critical care settings. Therefore, this study aims to investigate whether incorporating a standardized Check-list into resident training can achieve superior educational outcomes compared to traditional teaching methods, using the Mini-CEX as a formative and comprehensive assessment framework. By integrating the procedural standardization of the Check-list with the multidimensional evaluation strengths of the Mini-CEX, we aim to establish a balanced and effective training model suitable for high-acuity clinical environments.

## 2 Subjects and methods

### 2.1 Study objectives

#### 2.1.1 Study population

The 199 residents were enrolled in the study on a rolling basis between January 2018 and April 2024. The cohort consisted of 113 male and 86 female residents, with an average age of 29.69 ± 5.775 years. Each participant entered the study individually upon starting their emergency or critical care rotation and completed the control-phase Mini-CEX evaluation, followed by checklist-based training and post-intervention assessment. Therefore, the phrase “completed their training” refers to the completion of the study-specific teaching and evaluation process, not necessarily the formal end of their standardized residency training.

#### 2.1.2 Study methods

**FIGURE 1:**
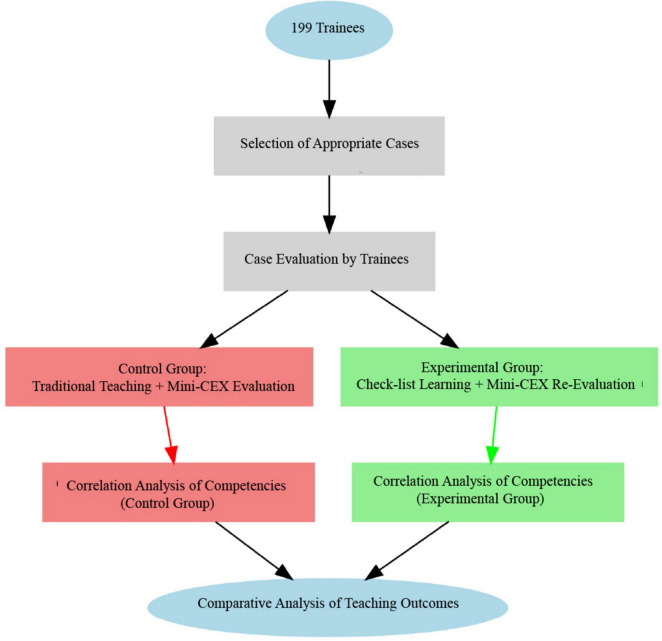
Flowchart of methods.

This study utilized the Mini-CEX as the primary outcome measure. The Mini-CEX assesses seven domains: medical interviewing skills, physical examination, humanistic qualities/professionalism, clinical judgment, consultation/advice/communication skills, organizational skills/efficiency, and overall clinical competence. Each domain was rated on a 9-point scale, where 1–3 indicated failure, 4–6 indicated pass, and 7–9 indicated excellence. The proportion of residents achieving each category was calculated based on the total sample (*n* = 199). In addition to the seven Mini-CEX domains, we also recorded each resident’s diagnosis and treatment time (hereafter referred to as “time”) as a quantitative measure of clinical efficiency. All evaluations were conducted by a fixed panel of three senior attending physicians, including the department’s teaching secretary and director of education. These assessors had completed formal training in competency-based assessment, in accordance with national residency evaluation protocols. Their fixed status throughout the study ensured inter-rater reliability and minimized subjective variability. The Check-list Scale, developed by the research team in accordance with contemporary emergency and critical care guidelines, expert consensus, and training standards, provided a structured framework to guide clinical performance during diagnostic and therapeutic processes. It included key domains such as history-taking, physical examination, diagnostic reasoning, treatment steps, and inter-professional communication. Residents in the control group first received traditional clinical training without the Check-list. Their performance was evaluated using the Mini-CEX following comparable clinical encounters. They then entered the intervention phase and received structured training based on the Check-list. Their performance was reassessed using the Mini-CEX on similarly matched clinical cases (Figure 1).

##### 2.1.2.1 Inclusion and exclusion criteria for participants

Inclusion criteria: (1) Junior residents who had not participated in similar teaching or assessment interventions within the past year; (2) Residents who completed both the control and intervention phases and had complete data records available.

Exclusion criteria: (1) Prior exposure to checklist-based training within the past year; (2) Incomplete participation in either phase or missing Mini-CEX data; (3) Obvious logical inconsistencies or outlier scoring patterns.

##### 2.1.2.2 Qualification criteria for assessors

(1) Held attending or senior academic titles; (2) Completed standardized rater training according to national guidelines; (3) Maintained fixed evaluator status throughout the study to ensure consistency; (4) Had no direct supervisory or mentoring relationship with the evaluated residents.

#### 2.1.3 Data analysis

**FIGURE 2:**
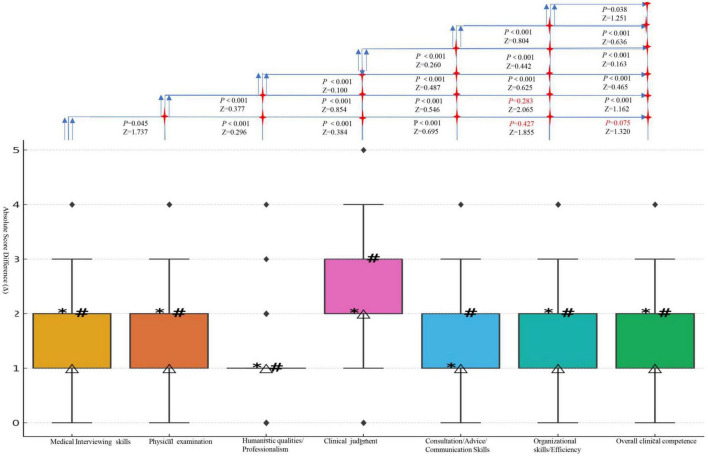
Boxplots of differences for each clinical skill; *, *M*_50_; △, *M*_25_; #, *M*_75_. The absolute score differences (Δ), ranked from highest to lowest, are as follows: clinical judgment: +2.417; physical examination: +2.407; organizational skills/Efficiency: +1.693; medical interviewing skills: +1.644; overall clinical competence: +1.543; consultation/advice/communication skills: +1.392; humanistic qualities/professionalism: +0.995. (All domains were confirmed to be non-normally distributed and were summarized using median and IQR. These raw score differences are for descriptive purposes only and should not be interpreted as statistically comparable across domains. Statistical significance should rely on *p*-values and Wilcoxon *Z*-statistics).

**FIGURE 3:**
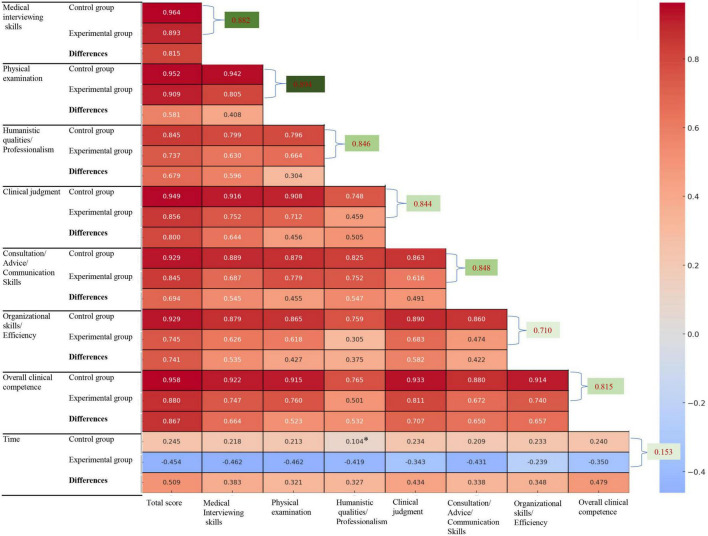
*Indicates *p* > 0.05. Correlation analysis of the same indicators before and after using Check-list is indicated in green.

**FIGURE 4:**
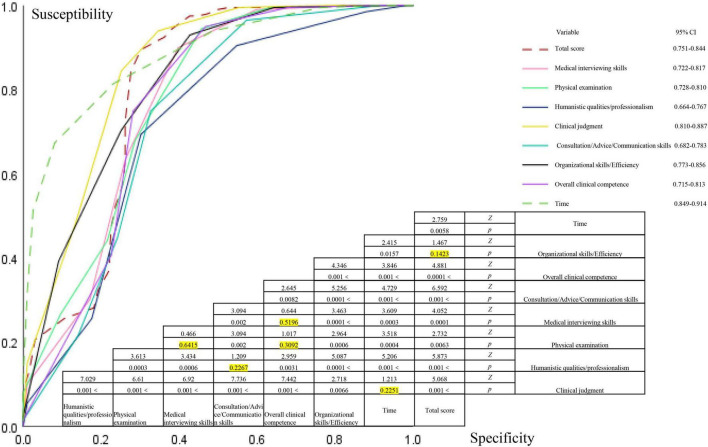
The receiver operating characteristic (ROC) curves of different indicators were compared pairwise, with yellow indicating a *p*-value greater than 0.05.

Categorical data were expressed as counts or percentages and analyzed using the chi-square test. Normally distributed data were presented as mean ± standard deviation (±s) and compared between groups using the *t*-test. Non-normally distributed data were analyzed using the rank-sum test. The correlation coefficient (ρ) between any two variables was calculated, and data analysis was performed using SPSS 20.0 software. The ROC curves were plotted and compared using MedCalc 22.0.3 software, with a significance level set at *p* < 0.05. Figures 2–4 were generated using R language.

## 3 Results

### 3.1 Comparison of Mini-CEX scores and treatment times between control and experimental groups

In this study, the Mini-CEX scoring method was used to evaluate 199 cases before and after the implementation of the Check-list. The comparison of various competency scores and classifications between the control and experimental groups is shown in Figures 2, 3 and Table 1.

**TABLE 1:** Comparison of Mini Clinical Evaluation Exercise (Mini-CEX) scores and time between control and experimental groups.

Variable	Control group	Experimental group	*P*-value	*Z/t/*χ^2^
**Total score**				
Fail, *n* (%)	5 (2.513)	0	< 0.001	27.332
Pass, *n* (%)	140 (70.352)	39 (19.598)	–	–
Excellent, *n* (%)	54 (27.136)	160 (80.402)	–	–
Score	34.000 (28.000, 46.000)	48.146 ± 6.629	< 0.001	−12.212
**Medical interviewing skills**				
Fail, *n* (%)	28 (14.070)	0	< 0.001	68.572
Pass, *n* (%)	119 (59.799)	73 (36.683)	–	–
Excellent, *n* (%)	52 (26.131)	126 (63.317)	–	–
Score	5.281 ± 1.715	6.925 ± 1.150	< 0.001	−26.143
**Physical examination**				
Fail, *n* (%)	73 (36.683)	1 (0.503)	< 0.001	107.771
Pass, *n* (%)	83 (41.709)	110 (55.276)	–	–
Excellent, *n* (%)	43 (21.608)	88 (44.221)	–	–
Score	4.000 (3.000, 6.000)	6.407 ± 1.414	< 0.001	−12.131
**Humanistic qualities/professionalism**				
Fail, *n* (%)	3 (1.508)	0	< 0.001	38.719
Pass, *n* (%)	136 (68.342)	61 (30.653)	–	–
Excellent, *n* (%)	60 (30.151)	138 (69.347)	–	–
Score	5.899 ± 1.330	6.894 ± 1.042	< 0.001	−19.653
**Clinical judgment**				
Fail, *n* (%)	40 (20.101)	0	< 0.001	112.944
Pass, *n* (%)	109 (54.774)	31 (15.578)	–	–
Excellent, *n* (%)	50 (25.126)	168 (84.422)	–	–
Score	5.000 (4.000, 6.500)	7.417 ± 1.065	< 0.001	−12.132
**Consultation/advice/communication skills**				
Fail, *n* (%)	16 (8.040)	0	< 0.001	83.521
Pass, *n* (%)	135 (67.839)	110 (55.276)	–	–
Excellent, *n* (%)	48 (24.121)	89 (44.724)	–	–
Score	5.000 (4.000, 6.000)	6.392 ± 1.175	< 0.001	−11.329
**Organizational skills/efficiency**				
Fail, *n* (%)	12 (6.030)	0	< 0.001	32.727
Pass, *n* (%)	137 (68.844)	59 (29.648)	–	–
Excellent, *n* (%)	50 (25.126)	140 (70.352)	–	–
Score	5.352 ± 1.434	7.045 ± 1.001	< 0.001	−23.634
**Overall clinical competence**				
Fail, *n* (%)	16 (8.040)	0	< 0.001	59.244
Pass, *n* (%)	127 (63.819)	50 (25.126)	–	–
Excellent, *n* (%)	56 (28.141)	149 (74.874)	–	–
Score	5.573 ± 1.640	7.116 ± 0.933	< 0.001	−18.776
Time	52.387 ± 7.635	66.985 ± 9.126	< 0.001	−18.776

#### 3.1.1 Score comparison between the two groups

Before the training, the overall failure rate was 2.513%, the pass rate was 70.352%, and the excellence rate was 27.136%. Among the various competencies, humanistic qualities/professionalism and overall clinical competence had the highest scores, while physical examination and clinical judgment had the lowest scores. After the training, the overall failure rate dropped to 0%, the pass rate decreased to 19.598%, and the excellence rate increased to 80.402%. At this stage, clinical judgment (5.899 ± 1.330) and overall clinical competence (52.387 ± 7.635) had the highest scores, while physical examination (median score: 4) and consultation/advice/communication skills (median score: 5) had the lowest scores (see Figure 2). The failure rates for all evaluation categories in the control group were significantly higher than those in the experimental group, while the rates of excellence in the experimental group were higher than those in the control group. The differences between the groups were statistically significant (*p* < 0.05). Specifically, in the experimental group, the pass rates for patient interview/medical communication skills, humanistic qualities/professionalism, clinical judgment, consultation/advice/communication skills, organizational skills/efficiency, and overall clinical competence were all lower than those in the control group, with statistically significant differences between the groups (*p* < 0.05) (Table 1).

#### 3.1.2 Differences before and after using the Check-list

After using the Check-list, there was a significant improvement in all competency scores (Table 1). The increases in scores for various competencies, ranked from highest to lowest, were as follows: clinical judgment > physical examination > organizational skills/efficiency > medical interviewing skills > overall clinical competence > humanistic care/professionalism > consultation/advice/communication skills (Figure 2). Except for the comparisons between medical interviewing skills vs. organizational skills/efficiency, medical interviewing skills vs. overall clinical competence, and physical examination vs. organizational skills/efficiency, all other pairwise comparisons showed statistically significant differences (*p* < 0.05) (Figure 2).

#### 3.1.3 Comparison of Mini-CEX scores and diagnosis/treatment times between the control and experimental groups

The control group had a median Mini-CEX score of 34.000 (IQR: 28.000, 46.000) and had an average time of 52.387 ± 7.635 min. In contrast, the experimental group had a significantly higher average Mini-CEX score of 48.15 ± 6.63 and had a longer time (66.985 ± 9.263 min). The differences between the two groups were statistically significant (*p* < 0.05), as detailed in Table 1.

### 3.2 Correlation analysis of different indicators

#### 3.2.1 Correlation of variables prior to the use of the scale

Correlation analysis showed that before using the Check-list, there was a strong correlation among the seven indicators assessed by the Mini-CEX (*p* < 0.05). Specifically, the correlation coefficients between the total Mini-CEX score and the medical interviewing skills, physical examination, and overall clinical competence were 0.964, 0.952, and 0.958, respectively (*p* < 0.001). The correlation between various indicators was high (ρ ≥ 0.50) too, whereas the correlation between time and each of the indicators was weak (ρ < 0.50) (Figure 3).

#### 3.2.2 Correlation among variables after the use of the scale

After using the Check-list, the correlations among the seven Mini-CEX indicators remained strong. The correlation coefficients between the total Mini-CEX score and medical interviewing skills, physical examination, and overall clinical competence were 0.893, 0.909, and 0.880, respectively (*p* < 0.001). However, the time showed a negative correlation with each of the indicators, though these correlations were weaker (*p* < 0.05, ρ < 0.50) (Figure 3).

#### 3.2.3 Pairwise comparison and correlation analysis of the differences in each indicator before and after using the scale

In the differences observed among the indicators after using the Check-list, the correlation coefficients between the total Mini-CEX score and medical interviewing skills, clinical judgment, and overall clinical competence were 0.815, 0.800, and 0.867, respectively (*p* < 0.001). The time was positively correlated with each of the indicators, with a strong correlation only with the total Mini-CEX score (ρ < 0.509); the correlations with other indicators were relatively weak (*p* < 0.05, ρ < 0.50) (Figure 3).

#### 3.2.4 Correlation analysis of the same indicators before and after using the scale

Except for the time indicator (*p* = 0.0313, ρ = 0.153), all other clinical competence-related indicators demonstrated a significant positive correlation before and after the use of the scale (*p* < 0.001). The correlations, ranked from highest to lowest, were as follows: total CEX score (0.945), physical examination (0.894), medical interviewing skills (0.882), advice/consultation/communication skill (0.848), humane qualities/professionalism (0.846), clinical judgment (0.844), overall clinical competence (0.815), and organizational skill and efficiency (0.710) (Figure 3).

### 3.3 ROC curve analysis

The ROC curve was used to perform sensitivity and specificity analysis based on the relationship between various indicators and scores before and after the use of the Check-list. The AUC for medical interviewing skills, physical examination, humanistic qualities/professionalism, clinical judgment, consultation/advice/communication skills, organizational skills/efficiency, overall clinical competence, Mini-CEX score, and time required were 0.770, 0.774, 0.716, 0.844, 0.733, 0.815, 0.764, 0.797, and 0.881, respectively, all indicating diagnostic efficacy (*p* < 0.05). These factors had a diagnostic efficacy on the total scores, as shown in Figure 4. Comparisons of the ROC curves for the seven indicators revealed that there was no statistically significant difference between overall clinical competence vs. clinical judgment, consultation/advice/communication skills, vs. humanistic qualities/professionalism, and physical examination vs. medical interviewing skills (*p* > 0.05). However, all other pairwise comparisons showed statistically significant differences (*p* < 0.05), as detailed in Figure 4.

## 4 Discussion

With soaring healthcare demand and increasingly stringent patient-safety standards (27), training competent emergency and critical-care physicians has become a worldwide challenge (28–30). In China, this problem is amplified because teaching is still dominated by lecture-based formats with little active or bedside learning (31–34). Our baseline evaluation quantifies this gap: only 27.136% of residents achieved an “excellent” rating, whereas > 20% failed key domains of clinical judgment and physical examination—skills indispensable for managing critically ill patients (35, 36). Several structural factors in China’s critical-care system further widen these deficits. First, critically ill patients often deteriorate rapidly (e.g., shock) and therefore require systematic diagnostic skills (37, 38), yet junior physicians struggle because of limited experience (39, 40). Second, compared with their Western counterparts, Chinese clinicians work under heavier pressures—from staffing shortages to complex workplace dynamics—leading to higher levels of anxiety, burnout, and other mental-health risks (41). Third, an over-emphasis on classroom teaching, an intensive research-productivity culture (42, 43), and rigid departmental management continually impede residents’ professional development (44, 45). Finally, although diagnostic technology has advanced, over-reliance on imaging and laboratory data is eroding foundational skills such as history-taking and physical examination (46–48); in complex critical illness this further elevates diagnostic-error risk (49). Collectively, these findings show that conventional instruction can no longer meet the demands of high-acuity care in China and underscore the need for a structured, competency-based intervention that combines check-list-guided standardization with Mini-CEX longitudinal assessment.

To address these challenges, educators have adopted tools such as the Mini-CEX to assess clinical competence (50). However, its standalone use remains insufficient to rapidly enhance junior physicians’ clinical judgment and therapeutic skills or reduce the high rate of medical errors in critically ill patients—estimated at 9 per 100 patients daily, with 10% deemed severe (51, 52)—largely due to limited experience, inadequate training, poor communication, and weak emergency responsiveness (53–55). Our results demonstrated that the checklist significantly improved scores across all assessed domain, standardizing clinical processes, and improving diagnostic efficiency (17–19). In this study, the introduction of checklists significantly improved performance across all assessed competencies, particularly in clinical judgment and physical examination (*p* < 0.05), further validating their value in clinical practice. This impact may stem from ICU physicians conducting structured assessments and treatment planning based on checklist steps, enabling even inexperienced doctors to follow protocols effectively under guideline-based supervision, thereby reducing the risk of misdiagnosis or oversight. Moreover, strong positive correlations were observed among various clinical competencies (*p* < 0.05), suggesting mutual reinforcement between them. ROC analysis further demonstrated that the checklist had the highest predictive accuracy for clinical judgment (AUC = 0.844) and organizational skills/efficiency (AUC = 0.815), likely reflecting its structured, guideline-driven design. Enhanced clinical judgment was also associated with improved performance in physical examination (AUC = 0.774) and medical interviewing skills (AUC = 0.770), which contributed to minimizing redundant procedures and diagnostic errors, ultimately improving overall clinical competence.

Although all competencies were improved through training, deficiencies remain in humanistic qualities/professionalism, as well as in consultation/advice/communication skills. These areas continue to pose significant challenges within the Chinese healthcare system. Our ROC curve analysis showed that improvements in humanistic qualities/professionalism and consultation/communication skills did not reach statistical significance (*p* > 0.05). This may reflect structural flaws in the current evaluation system: (many existing checklists use binary (“yes” or “no”) formats and lack graded criteria or practical guidance, a limitation noted in prior studies (51–59). Deeper systemic factors further exacerbate this problem. Frequent medical disputes (57) have led to the widespread adoption of defensive medical practices. Simultaneously, reforms such as the Diagnosis-Related Group (DRG) payment system (58) and healthcare cost-control policies (41) have significantly limited the time and energy available for clinicians to cultivate humanistic qualities. Moreover, persistent clinical and administrative burdens (54) have contributed to widespread occupational burnout, eroding professional identity and perpetuating a vicious cycle of reduced empathy and job dissatisfaction (60). This “institutional pressure–behavioral distortion–competency degradation” cascade not only hampers the development of humanistic values among medical trainees but also undermines the quality of clinical education. Given its deeply rooted institutional nature, this issue cannot be effectively resolved through technical interventions alone.

Some studies have shown that the use of checklists may lead to longer diagnostic and treatment times (17), which is consistent with the findings of this study. Our results indicate that the application of the checklist significantly increased the time (66.985 ± 9.126 vs. 52.387 ± 7.635 min). Although this may superficially suggest a decline in clinical efficiency, the overall comparison of overall clinical competence scores indicates an actual improvement in efficiency. This improvement may be attributed to the checklist’s ability to enhance the accuracy and standardization of physical examination, medical interviewing skills, and treatment procedures, thereby reducing omissions and errors (18). If feasible, future studies should further investigate the relationship between diagnostic/treatment duration, clinical efficiency, and competency outcomes through large-scale, multicenter clinical trials.

In conclusion, this study used the Mini-CEX as a validation tool to assess whether checklist-guided instruction is superior to traditional training in improving residents’ clinical competence. Our findings demonstrate that combining the procedural rigor of checklists with the formative, holistic evaluation of Mini-CEX offers a promising training model. This dual approach not only enhances key clinical skills—especially clinical judgment and efficiency—but also strengthens emergency response and bridges the gap between theoretical knowledge and practical application. However, ongoing adaptation is needed to address non-technical skill gaps and meet evolving healthcare demands.

## 5 Limitations

(1) The study may have focused only on the short-term effects of the training without tracking the long-term improvement and stability of clinical skills. (2) External factors such as hospital resources, the baseline competency levels of resident physicians, and the educational environment may have influenced the training outcomes but were not fully explored in this study. (3) The study was conducted in China, and cultural and regional differences might affect the generalizability of the results internationally. Different healthcare systems and educational approaches in other countries may require alternative evaluation tools or training methods. (4) Although the ROC curve analysis provided data on diagnostic efficacy, the direct impact on actual clinical outcomes (such as patient outcomes) was not assessed. (5) The COVID-19 pandemic (2020–2022) affected the availability of residents for enrollment due to changes in clinical rotations and staffing demands. This resulted in a slower recruitment rate and extended the overall study duration. However, the training design, assessment procedures, and quality standards were consistently maintained throughout the study period.

## Data Availability

The original contributions presented in this study are included in this article/supplementary material, further inquiries can be directed to the Corresponding authors.
